# Characterization of the GH13 and GH57 glycogen branching enzymes from *Petrotoga mobilis* SJ95 and potential role in glycogen biosynthesis

**DOI:** 10.1371/journal.pone.0219844

**Published:** 2019-07-15

**Authors:** Xuewen Zhang, Hans Leemhuis, Marc J. E. C. van der Maarel

**Affiliations:** 1 Department of Aquatic Biotechnology and Bioproduct Engineering, Engineering and Technology institute Groningen, University of Groningen, Groningen, Netherlands; 2 Avebe Innovation Center, Groningen, Netherlands; Weizmann Institute of Science, ISRAEL

## Abstract

Glycogen is a highly branched α-glucan polymer widely used as energy and carbon reserve by many microorganisms. The branches are introduced by glycogen branching enzymes (EC 2.4.1.18), that are classified into glycoside hydrolase families 13 (GH13) and 57 (GH57). Most microorganisms have typically only a single glycogen branching enzyme (*gbe*) gene. Only a few microorganisms carry both GH13 and GH57 *gbe* genes, such as *Petrotoga mobilis* and *Mycobacterium tuberculosis*. Here we report the basic characteristics of the GH13 and GH57 GBE of *P*. *mobilis*, both heterologously expressed in *E*. *coli*. The GH13 GBE has a considerably higher branching activity towards the linear α-glucan amylose, and produces a highly branched α-glucan with a high molecular weight which is very similar to glycogen. The GH57 GBE, on the contrary, makes a much smaller branched α-glucan. While the GH13 GBE acts as a classical glycogen branching enzyme involved in glycogen synthesis, the role of GH57 GBE remains unclear.

## Introduction

The phylum *Thermotogae* is currently composed of 50 species spread across 13 genera. All its members have a characteristic outer membrane that lies loosely around the cells. The genera are grouped into 5 families [1, 2]: (i) *Fervidobacteriaceae*, comprising the genera *Fervidobacterium* [1] and *Thermosipho* [3]; (ii) *Kosmotogaceae*, comprising the genera *Kosmotoga* [4] and *Oceanotoga*; (iii) *Mesoaciditogaceae*, comprising the genera *Mesoaciditoga* [5, 6] and *Athalassotoga*; (vi) *Petrotogaceae*, comprising the genera *Petrotoga* [7], *Defluviitoga* [8], *Geotoga* [7], *Marinitoga* [9] and *Oceanotoga* [10]; and (v) *Thermotogaceae*, comprising the genera *Thermotoga* and *Pseudothermotoga* [2]. These thermophilic bacteria live in hot, anaerobic environments, such as hot springs, the deep-sea floor, or oil reservoirs. Thirty-six genome sequences of members of the *Thermotogae* phylum have been reported in the last few years covering all five families. All the genomes contain the key enzymes for glycogen synthesis, namely ADP-glucose pyrophosphorylase (GlgC, EC 2.7.7.27), glycogen synthase (GlgA, EC 2.4.1.11), and glycogen branching enzyme (GlgB, EC 2.4.1.18), together catalyzing the conversion of glucose-1-phosphate into glycogen [11].

Glycogen is an intracellular α-glucan reserve polymer of many microorganisms and eukaryotes [11]. It is composed of anhydroglucose residues with approximately 90% α-1,4-glycosidic linkages and branched by approximately 10% α-1,6-glycosidic linkages. In many bacteria, glycogen is the major reserve polymer accumulated during exponential growth [11]. Besides an energy reserve, glycogen can have other functions in bacteria: as a protectant of proteins and membranes [12], as a transcriptional regulator [13], as a structural constituent in the cell wall [14], and as a regulatory molecule in glucose metabolism [15]. Glycogen branching enzyme (GBE), being one of the key enzymes in glycogen synthesis, cleaves an α-1,4-glycosidic linkage in a growing α-1,6 glucan chain and subsequently attaches the cleaved-off fragment onto the C6 hydroxyl of the anhydroglucose moiety within an α-1,4 glucan chain [11, 16] *i*.*e*. the branch. All GBEs known so far are found in the glycoside hydrolase families 13 (GH13) and 57 (GH57) [17, 18].

Whereas most bacteria only have either a GH13 or a GH57 GBE, some bacteria have two GBE encoding genes, one GH13 and one GH57 [19]. An example of this is *Mycobacterium tuberculosis*, the causative agent of tuberculosis, a disease killing more than one million people each year, which has a GH13 and a GH57 GBE [20, 21]. The genomes of different *M*. *tuberculosis* strains have been sequenced and almost all these strains contain both a GH13 and a GH57 *gbe* indicating that both enzymes are required (21). Deletion of the GH13 *gbe* (Rv1326c) of *M*. *tuberculosis* did not give viable mutants, indicating an essential role for the GH13 GBE [22]. The putative GH57 GBE (Rv3031) has only been predicted from protein sequence homology [20], and has so far not been characterized; it has been proposed that this protein plays a role in lipopolysaccharide synthesis [23, 24].

In the genomes of the members of the *Petrotogaceae*, except *Geotoga petraea*, *Petrotoga mexicana*, and *Petrotoga sibirica*, two *gbe* genes are present, one encoding a GH13 GBE and one a GH57 GBE. Several GH13 and GH57 GBEs have been characterized in detail [20, 21, 25–28]. However, characterization of the GH13 and GH57 GBEs from a single species has not been reported and thus it is not clear if both GBEs play a role in glycogen synthesis or only one of the two, as is suggested for *M*. *tuberculosis*. In this work, the genes encoding the putative GH13 and GH57 GBE from *P*. *mobilis* were overexpressed and both enzymes were shown to be functional GBEs *in vitro*. PmGBE13 (GH13) shows 130 folds higher activity than PmGBE57 (GH57) with long chain amylose V as substrate. These two GBEs also differ in the degree of branching of the final branched α-glucans; the PmGBE57 produces an 8.5% branched product while the PmGBE13 makes a 12.4% branched product with more short chains.

## Materials and methods

### Materials

Amylose V was provided by Avebe (Veendam, Netherlands). Lithium bromide was obtained from Acros Organics. Isoamylase (EC 3.2.1.68, specific activity 260 U/mg), pullulanase M1 (EC 3.2.1.41, specific activity 34 U/mg) and β-amylase (EC 3.2.1.2, specific activity 10,000 U/mL) were obtained from Megazyme (Wicklow, Ireland). DHBS was obtained by debranching highly branched starch (HBS, 8% α-1,6-linkages) with isoamylase and pullulanase; HBS was obtained by modifying gelatinized potato starch with the Branchzyme, an enzyme preparation produced by Novozymes (Bagsvaerd, Denmark) containing the glycogen branching enzyme from *Rhodothermus obamensis*. The oligosaccharide kit was purchased from Sigma-Aldrich (Zwijndrecht, Netherlands).

### Sequence collection

The sequences of GH13 GBEs from *Thermotoga* bacteria were collected based on the preliminary analysis of family GH13_9, from the CAZy database [29]. The sequences of GH57 GBEs were collected based on a BLAST search using the complete sequences of the specified GBEs from *Thermus thermophilus* (UniProt accession no. Q5SH28) and *Thermococcus kodakarensis* KOD1 (UniProt accession no. Q5JDJ7) with the genome sequences of *Thermotoga* bacteria. All the selected potential sequences had to possess the GH57 characteristic signatures, such as five conserved sequence regions (CSRs), both catalytic residues and a (β/α)_7_ barrel domain [18].

### Expression and purification of PmGBE13 and PmGBE57

Codon optimized genes (*glgB13* and *glgB57*) encoding the GH13 (PmGBE13) and GH57 (PmGBE57) GBE of *P*. *mobilis* SJ95 were synthesized by GenScript, and cloned into the *Nde*I-*Bam*HI sites of the pET28a vector (Novagen), with a 6×His-tag fused at the C-terminal. Sequence details are provided in the supplemental information. *glgB13* and *glgB57* were overexpressed in *Escherichia coli* BL21(DE3) cultivated in Luria-Bertani (LB) medium (10 g/L of tryptone, 5 g/L yeast extract, and 10 g/L NaCl) supplemented with 50 μg/mL kanamycin at 16°C for 20 h and 150 rpm. Cells were harvested by centrifugation (5,000×*g*, 10 min, 4°C). Cells were washed twice with 10 mM sodium phosphate buffer pH 7.0 and lysed by high-pressure homogenizer (Emulsiflex-B15; Avestin). The cell free extract was collected by centrifugation (20,000×*g*, 20 min, 4°C). PmGBE13 and PmGBE57 were purified in two steps. Firstly, the cell free extracts were heated at 65°C for 20 min, followed by removal of the denatured proteins by centrifugation (20,000×*g*, 20 min, 4°C). Subsequently, the His-tagged proteins were purified using HisPurTM Ni-NTA Resin (ThermoFisher Scientific) according to the manufacturer’s protocol. Protein concentration was quantified using the Quick Start Bradford Protein Assay kit (Bio-Rad Laboratories). Purity and molecular mass of the proteins were checked by SDS-PAGE.

### Enzyme activity assays

Activity of PmGBE13 and PmGBE57 was measured with amylose V as substrate. Reaction progress was followed by iodine staining, which is based on monitoring the decrease of the absorbance of the glucan-iodine complex [30]. Amylose V was dissolved in 1 M sodium hydroxide, and then neutralized to pH 7.0 with 1 M HCl. The reaction mixture consisted of 0.125% (w/v) amylose V in 50 mM sodium phosphate buffer (pH 7.0). Reactions were performed at 50°C and started by the addition of enzyme, 3 μg/mL PmGBE1 or 60 μg/mL PmGBE2. Reaction progress was followed in time by taking 10 μL aliquots and adding them to 150 μL iodine reagent (aqueous solution of 0.0127% I_2_ (w/v) and 0.035% KI (w/v)). The absorption at 660 nm was measured. One unit of activity is defined as the decrease in absorbance of 1.0 per min at 660 nm.

The total activity, being the sum of hydrolytic and branching activity, was determined by measuring the reducing ends of the product after debranching by isoamylase and pullulanase. The reducing ends were measured by the BCA method [31]. Prior to debranching the products, enzymes were inactivated by incubating the samples at 100°C for 10 min. One unit of total activity is defined as 1 μmol total reducing ends synthesized per minute.

Branching activity, representing the newly synthesized α-1,6-glycosidic linkages, was determined by measuring the increase in reducing ends before and after debranching of the product by isoamylase and pullulanase. One unit of branching activity is defined as 1 μmol of α-1,6-linkage synthesized per minute. The data used to calculate different activities were from 0 to 30 min of incubation.

The influence of temperature and pH on the activity of PmGBE13 and PmGBE57 was tested in 50 mM sodium phosphate buffer at pH 7.0 or 50°C, respectively. The temperature ranged from 40 to 80°C and the pH from 6.0 to 9.0. Amylose V at a concentration of 0.125% (w/v) was used as substrate and the activity was quantified using the iodine staining assay.

### High performance anion exchange chromatography

Oligosaccharide analysis was carried out by High Performance Anion Exchange Chromatography (HPAEC) on a Dionex ICS-3000 system (Thermo Scientific, USA) equipped with a 4×250 mm CarboPac PA-1 column. A pulsed amperometric detector with a gold electrode and an Ag/AgCl pH reference electrode were used. The system was run with a gradient of 30–600 mM NaAc in 100 mM NaOH run at 1 mL/min. Chromatograms were analyzed using Chromeleon 6.8 chromatography data system software (Thermo Scientific). A mixture of glucose, maltose, maltotriose, maltotetraose, maltopentaose, maltohexaose and maltoheptaose (0.1 mg/mL of each component) was used as reference for qualitative determination of elution time of each component. The decay rate of detector signal from DP 2 to DP 7 calculated from reference sample is 4.44:2.76:2.02:1.45:1.36:1.00, which is used to correct the DP 2 to DP 7 of all samples and other components are without correction.

### ^1^H-NMR spectroscopy

^1^H-NMR spectra were recorded at a probe temperature of 323 K on a Varian Inova 500 spectrometer (NMR Center, University of Groningen). All samples produced by PmGBE1 or/and PmGBE2 were dialyzed by dialysis tubing with cutoff size of 100 to 500 Da. Subsequently, all samples were freeze dry. Before analysis, samples were exchanged twice in D_2_O (99.9 atom% D, Sigma-Aldrich Chemical) with intermediate lyophilization, and then dissolved in 0.6 mL D_2_O. Spectra were processed using MestReNova 5.3 software (Mestrelabs Research SL, Santiago de Compostella, Spain), using Whittaker Smoother baseline correction and zero filling to 32 k complex points. Chemical shifts (δ) are expressed in ppm by reference to internal acetone (δ 2.225 for ^1^H). Carbohydrate structures were determined using the previously developed ^1^H-NMR structural-reporter-group concept of α-D-glucans [32]. The α-1,6 signal is presented at δ 4.98, originating from H1 in 1,4-α-glucose-1,6 and α-1,4 signal is at δ 5.36 from the H1 in 1,4-α-glucose-1,4 and 1–4,6-α-glucose-1,4 residues.

### Molecular weight distribution analysis

Molecular weight distributions were measured by Gel Permeation Chromatograph (GPC). DMSO-LiBr (0.05 M) was prepared by stirring 3 h at room temperature. Samples were dissolved at a concentration of 2 mg/mL in DMSO-LiBr at 80 ^o^C with shaking for 3h and filtered through a 0.45 μm Millex PTFE membrance (Millipore Corporation, Billerica, USA). The Size Exclusion Chromatography (SEC) system setup (Agilent Technologies 1260 Infinity) from PSS (Mainz, Germany) consisted of an isocratic pump, auto sampler without temperature regulation, an online degasser, an inline 0.2 μm filter, a refractive index detector (G1362A 1260 RID Agilent Technologies), viscometer (ETA-2010 PSS, Mainz), and MALLS (SLD 7000 PSS, Mainz). WinGPC Unity software (PSS, Mainz) was used for data processing. The samples were injected with a flow rate of 0.5 mL min^-1^ into a PFG guard-column and three PFG SEC columns 100, 300 and 4000, which were also purchased from PSS. The columns were held at 80°C, the visco-detector at 60°C (Visco) and the RI detector at 45°C (RI). A universal calibration curve was generated using a standard pullulan kit (PSS, Mainz, Germany) with molecular weights from 342 to 805,000 Da, in order to determine the hydrodynamic volume from the elution volume. The specific RI increment value dn/dc was measured by PSS and is 0.072.

### Chain length analysis

The structure of a branched α-glucan can be described by the average length of the linear chains in total (ACL) and the average length of the linear chains between two branch points, i.e. the internal chain length (ICL). The ACL was calculated from peak area of HPAEC profiles. Based on the chain length distribution three fractions were classified as DP 2–4, DP 5–10 and DP above 10. The percentage of each fraction was calculated from the peak area. In order to reduce the influence of attenuate signal, the attenuate ratio was tested by standard sample containing the same mass concentration fractions glucose, maltose, maltotriose, maltotetraose, maltopentaose, maltohexaose, and maltoheptaose.

Based on the relations of one chain with others the chains of α-glucan are classified into three types [33, 34]: the A chain is linked only through its reducing terminus to carbon 6 of a glucose unit of another chain; the B chain is linked at its reducing end to another B or to a C chain while at the same time it carries one or more A and/or B chains as branches; the C chain is the chain with the only free reducing end in the molecule. The internal chain length (ICL) is defined as the average number of glucose units between two branching points in B chains. The ICL is determined by first trimming the exterior chains using the exo-acting enzyme β-amylase. A-chains are shortened to 2 or 3 anhydroglucose units and, for B-chains to 1 or 2 anhydroglucoses [35]. Briefly, branched α-glucan products (2 mg) were treated with 10 units β-amylase at 40°C in 5 mM sodium citrate buffer pH 6.5 for 24 h. β-amylase was inactivated by boiling for 5 min. Subsequently the pH was lowered to 5.0 with citric acid, followed by overnight isoamylase and pullulanase debranching at 40°C. The debranched samples were analyzed by HPAEC. The chain length distribution was then compared to the distribution of the samples without β-amylase treatment, as described. The percentage of A-chains was calculated as the ratio between two folds peak area of maltotriose (A-chains were hydrolyzed to maltose and maltotriose by β-amylase) and total peak area. The AICL was calculated as follow:

$$AICL=\frac{BAM-1.5}{100/(100-A\%)}-1$$

BAM: the average chain length of β-amylase treated α-glucans was calculated from peak area of DP≥4 in HPAEC spectra. A%: the percentage of A-chains in α-glucans.

## Results and discussion

### Distribution of genes encoding glycogen branching enzymes in *Thermotoga* species

GBEs are key enzymes in the synthesis of glycogen, a reserve polymer of many microorganisms, invertebrates, and animals [36]. In this study the distribution of GBEs in 42 *Thermotoga* species, of which the whole genome sequence has been published, is reported. All these 42 *Thermotoga* species have one or two glycogen branching enzyme genes, a glycogen synthase gene, and a glucose-1-phosphate adenylyltransferase gene, indicating that they all have the capacity to synthesize glycogen (Table 1). All Thermotogaceae, Fervidobacteriaceae, Kosmotogaceae, and Mesoacidotogaceae have a single GH57 *glgB*. Within the Petrotogaceae, *Geotoga petraea* and *Petrotoga sibirica* possess a single GH13 *gbe* while *Petrotoga hypogea* has two GH57 *gbe*s (Table 1). *Defluviitoga tunisiensis*, *Marinitoga* sp., *M*. *hydrogenotolerans*, *M*. *peizophila*, *Petrotoga mobilis*, *Petrotoga halophile*, and *P*. *miotherma* are remarkable as they possess both a GH13 and a GH57 *gbe*. The presence of a GH13 and putative GH57 *gbe* in one and the same species raises the question whether the corresponding GBEs are functional and if they both play a role in glycogen biosynthesis. To shed some light on this question, the GH13 and GH57 GBE genes and corresponding enzymes of *P*. *mobilis* were studied in more detail.

**Table 1:** Occurrence of the key enzymes in glycogen synthesis in members of *Thermotogacea*. GBE: glycogen branching enzyme; GSE: glycogen synthase; AGPase: ADP-glucose pyrophosphorylase.

Families	Name	GH13 GBE	GH57 GBE	GSE	AGPase
Thermotogaceae	*Thermotoga caldifontis* AZM44c09		WP_041077987.1	WP_041077756.1	WP_041075563.1
	*Thermotoga maritima* MSB8		WP_004081707.1	NP_228703.1	NP_228053.1
	*Thermotoga naphthophila* RKU-10		WP_012896461.1	WP_011942738.1	WP_012896196.1
	*Thermotoga neapolitana* DSM 4359		WP_038067483.1	ACM23857.1	ACM22623.1
	*Thermotoga petrophila* RKU-1		WP_011943829.1	WP_011942738.1	WP_012310694.1
	*Thermotoga profunda* AZM34c06		WP_041082268.1	WP_041082835.1	WP_041083424.1
	*Thermotoga sp*. 2812B		WP_008195099.1	WP_004080686.1	WP_004082940.1
	*Pseudothermotoga elfii* DSM 9442		WP_012003808.1	WP_012003430.1	WP_028843492.1
	*Pseudothermotoga hypogea* DSM 11164		WP_031503953.1 WP_081836282.1	WP_031504818.1	WP_031504508.1
	*Pseudothermotoga lettingae* TMO		WP_012003808.1	WP_012003430.1	WP_012002332.1
	*Pseudothermotoga thermarum* DSM 5069		WP_013933073.1	WP_013933165.1	WP_013932574.1
Fervidobacteriaceae	*Fervidobacterium islandicum *DSM 17883		WP_033191873.1	WP_033191209.1	WP_033191528.1
	*Fervidobacterium gondwanense* DSM 13020		WP_072759036.1	WP_072760135.1	WP_072757324.1
	*Fervidobacterium nodosum* Rt17-B1		WP_011994035.1	WP_011993493.1	WP_011994654.1
	*Fervidobacterium pennivorans* DSM 9078		WP_041262849.1	WP_014451068.1	WP_014450695.1
	*Fervidobacterium thailandensis *		WP_069293104.1	WP_069293649.1	WP_069292357.1
	*Thermosipho affectus*		WP_075665539.1	WP_077197850.1	WP_075665454.1
	*Thermosipho africanus* H17ap60334		WP_012579629.1	WP_012579478.1	WP_012579605.1
	*Thermosipho africanus* Ob7		WP_114702187.1	WP_114702310.1	WP_114702215.1
	*Thermosipho africanus* TCF52B		WP_012579629.1	WP_012579478.1	WP_012579604.1
	*Thermosipho atlanticus* DSM 15807		WP_073073190.1	WP_073072419.1	WP_073072127.1
	*Thermosipho globiformans*		WP_126992764.1	WP_126992938.1	WP_126993134.1
	*Thermosipho melanesiensis* BI429		WP_012056570.1	WP_012056316.1	WP_012056477.1
	*Thermosipho sp*. 1063		WP_008195099.1	WP_075665323.1	WP_075665454.1
Petrotogaceae	*Defluviitoga tunisiensis* DSM 23805	WP_045087168.1	WP_045087488.1	WP_045087536.1	WP_045087221.1
	*Geotoga petraea* ATCC 51226	WP_091403612.1		WP_091405813.1	WP_091402621.1
	*Marinitoga hydrogenitolerans* DSM 16785	WP_072864394.1	WP_072862856.1	WP_072863441.1	WP_072864199.1
	*Marinitoga piezophila* KA3	WP_014296184.1	WP_014297024.1	WP_014296945.1	WP_014296021.1
	*Marinitoga sp*. 1155	WP_047265238.1	WP_047265848.1	WP_075780662.1	WP_075780313.1
	*Petrotoga mobilis* SJ95	WP_012209120.1	WP_012208426.1	WP_012208925.1	WP_012208734.1
	*Petrotga halophila* DSM 16923	WP_103898766.1	WP_103898294.1	POZ92479.1	WP_012208734.1
	*Petrotoga mexicana* DSM 14811		WP_103077822.1	PNR98227.1	PNR98748.1
	*Petrotoga miotherma* DSM 10691	WP_103079200.1	WP_103078532.1	PNS02142.1	PNS02470.1
	*Petrotoga olearia* DSM 13574	WP_103066081.1	WP_103066988.1	PNS02142.1	PNS02470.1
	*Petrotoga sibirica* DSM 13575	WP_103876993.1		WP_103876724.1	WP_103876533.1
Kosmotogaceae	*Kosmotoga arenicorallina* S304		WP_068347510.1	WP_068347125.1	WP_068346683.1
	*Kosmotoga olearia* TBF 19.5.1		WP_012744890.1	WP_015869246.1	WP_015868560.1
	*Kosmotoga pacifica*		WP_047754759.1	WP_047755003.1	WP_047754440.1
	*Kosmotoga sp*. DU53		WP_012744890.1	WP_015869246.1	WP_015868270.1
	*Mesotoga infera *		KUK67992.1	CCU85914.1	CCU83670.1
	*Mesotoga prima* MesG1.Ag.4.2		WP_006486989.1	WP_014730450.1	WP_006486752.1
Mesoaciditogaceae	*Mesoaciditoga lauensis* DSM 25116		WP_036226301.1	WP_036221777.1	WP_036226373.1

The gene *pmgbe13* (encoding the GH13 GBE of *P*. *mobilis*) has a complete ORF with a start codon at position 1,405,056 and stop codon at position 1,407,239 in the genome sequence. A clear promoter was predicted in the upstream region (2,000 bp) of the *pmgbe13* gene with a clear -10 (TTTTATAAT) and -35 (TTTAAA) consensus sequence (http://www.softberry.com/). *pmgbe57* (encoding the GH57 GBE of *P*. *mobilis*) also has a complete ORF from position 639,260 to position 640,876 in the genome sequence. The promoter was predicted in the upstream region (2,000 bp) with a -10 (CTCTACTAT) and -35 (TTTAAT) consensus sequence. Several transcription factor-binding sites were predicted in the upstream sequences for both genes. These results taken together indicate that both genes are functional and can be translated and regulated *in-vivo*. As expected for GBEs no signal sequences were identified, suggesting that these two GBEs are not excreted and are active intracellularly. As the *P*. *mobilis* genome sequence also contains a glycogen synthase and a glycogen debranching enzyme, it is concluded that *P*. *mobilis* contains all the key enzymes for glycogen synthesis making it likely that it synthesizes glycogen. So far no studies on the presence and structure of the glycogen from any of the *Petrotogaceae* have been reported.

### Biochemical properties of PmGBE13 and PmGBE57

**Fig 1:**
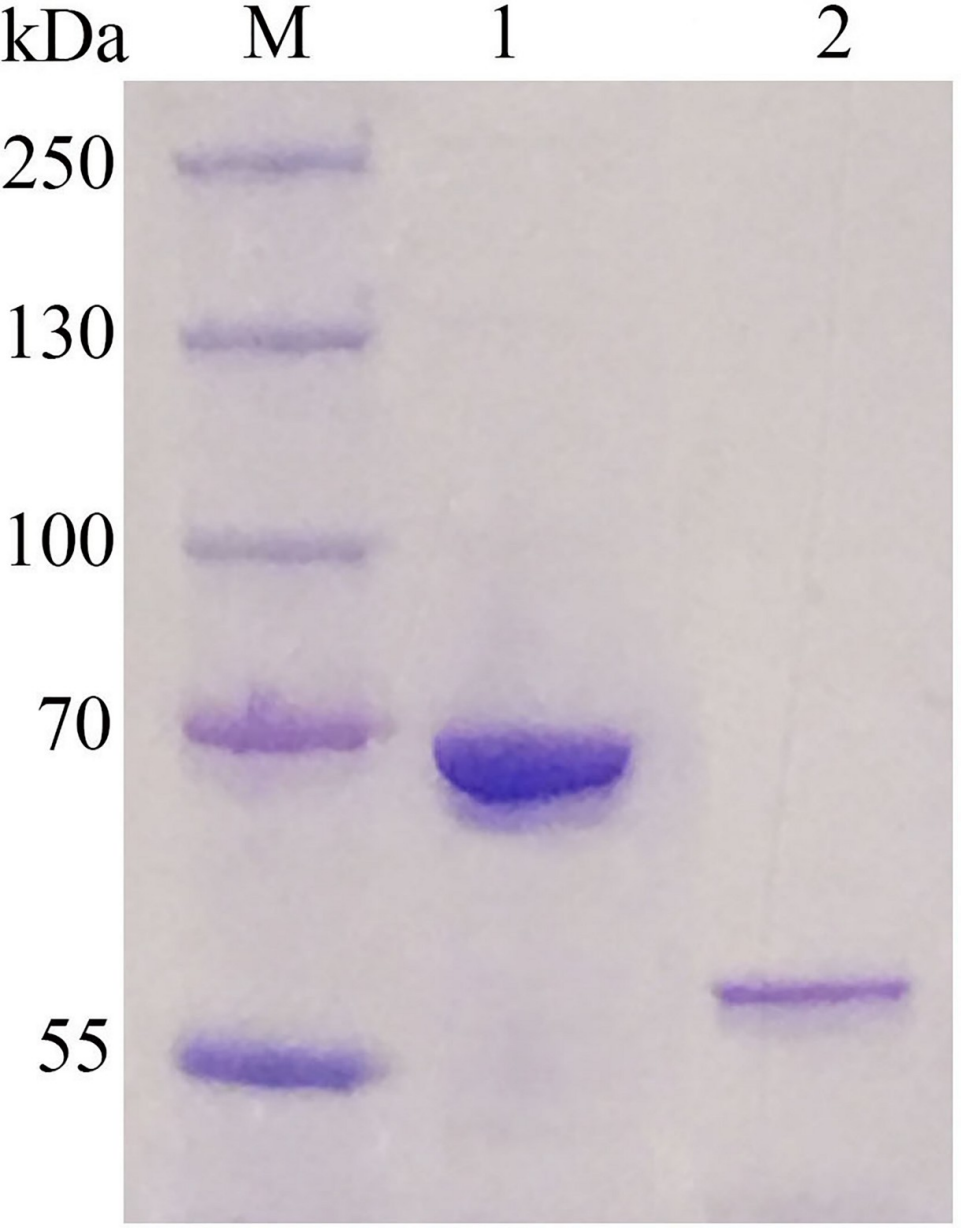
SDS-PAGE of purified PmGBE13 (lane 1) and PmGBE57 (lane 2). M: protein standard.

**Fig 2:**
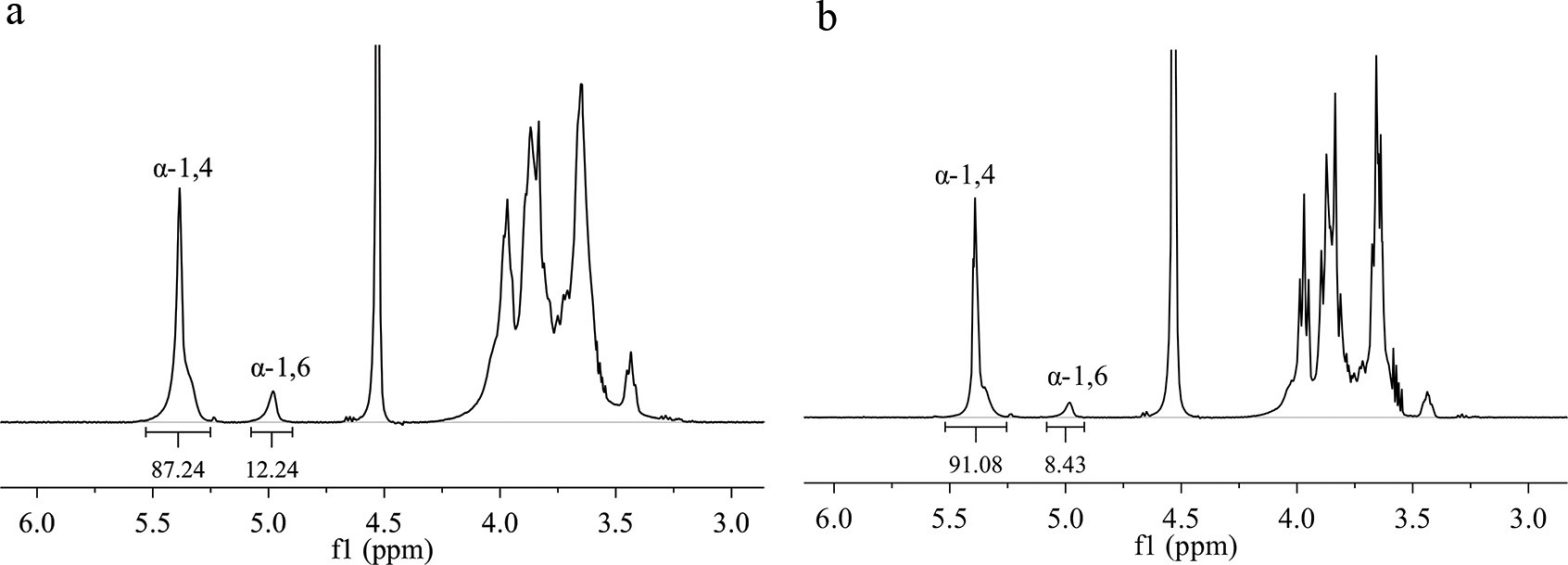
**^1^H-NMR spectrum of the branched α-glucans derived from amylose V by the action of PmGBE13 (a) and PmGBE57 (b).** Reactions were performed in phosphate buffer pH 7.0 at 50°C for 24 h. The spectra were recorded in D_2_O at 323 K. The signal originated by the residual water in the sample (HOD peak at 4.24 ppm) was cut off from the spectrum.

To investigate if the putative PmGBE13 and putative PmGBE57 have glycogen branching activity, the corresponding genes were over expressed in *E*. *coli* BL21(DE3). The obtained enzymes were purified to homogeneity, as judged by SDS-page (Fig 1). Both purified proteins convert amylose V at 60°C and pH 7.0, as revealed by a decrease in iodine staining, thus demonstrating that both putative GBEs are functional α-glucan modifying enzymes. Subsequent ^1^H-NMR analysis demonstrated that both enzymes convert amylose V in branched α-glucans with a degree of branching of 12.4% for the PmGBE13 and 8.5% for the PmGBE57 (Fig 2). The branching degree of the branched α-glucan products of PmGBE57 is in the range of those found for other GBEs [37–39]. The branching degree of the PmGBE13 product is one of the highest values reported so far, being in the same range as the branched α-glucan product (13.5%) made by the *Geobacillus thermoglucosidans* GBE with amylose as substrate [40]. The PmGBE13 enzyme is an interesting enzyme to further explore as it is not only thermostable, which is an advantage in starch processing as this is done at temperatures above 60 ^o^C [41], but also the high degree of branching of the products could contribute to a slower digestion in the small intestine, possibly making this branched α-glucan a slow digestible starch [37, 42, 43].

**Fig 3:**
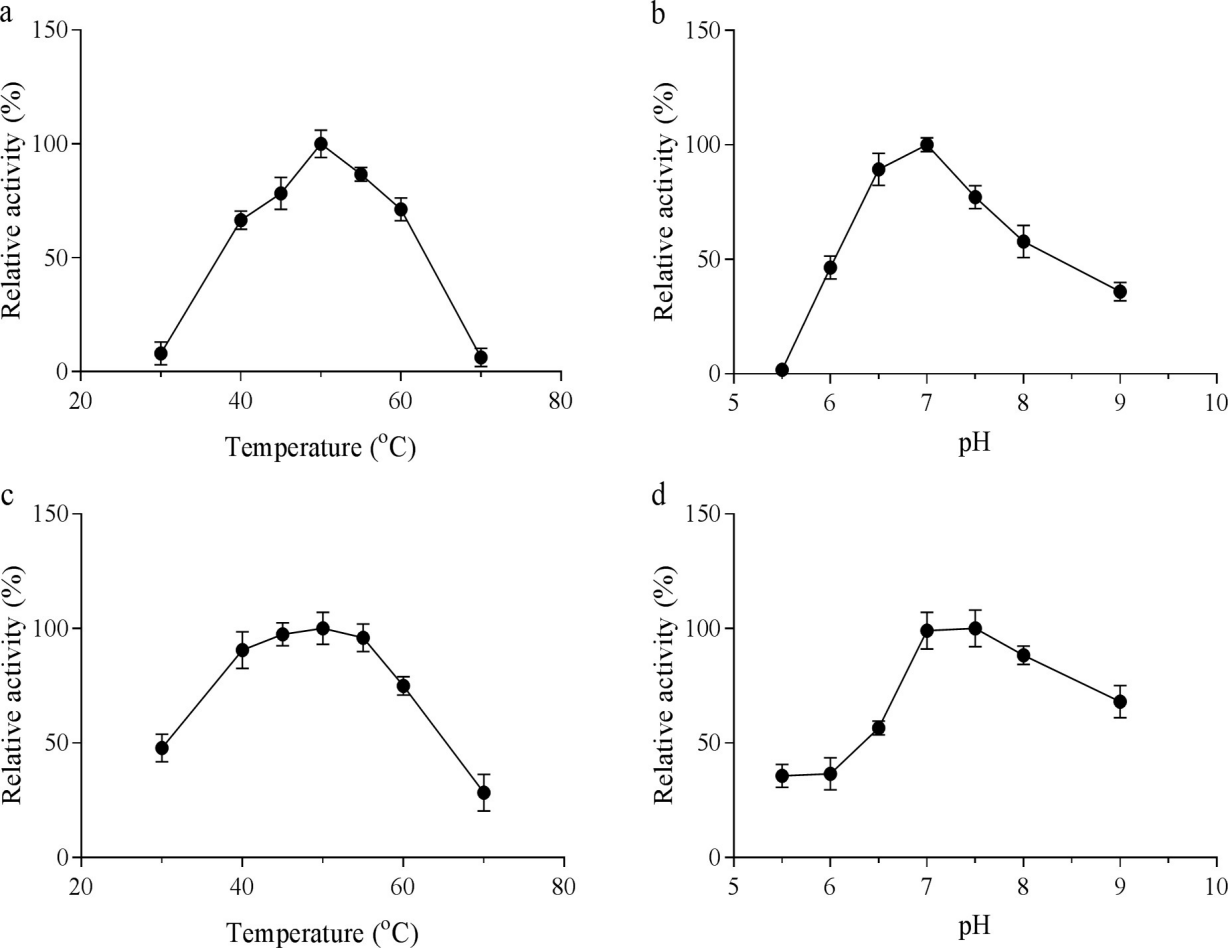
**Temperature and pH activity profiles of PmGBE13 (a, b) and PmGBE57 (c, d)**.

The influence of temperature and pH on the activity of PmGBE1 and PmGBE2 was investigated. Both enzymes showed maximum activity at 50°C and pH 7.0, and lost activity at temperatures of 70°C and higher (Fig 3). Of the two enzymes, the activity of PmGBE13 is most sensitive to changes in temperature and pH. The maximum activity at neutral pH also supports the earlier conclusion that both enzymes are very likely to be active in the cytosol.

**Fig 4:**
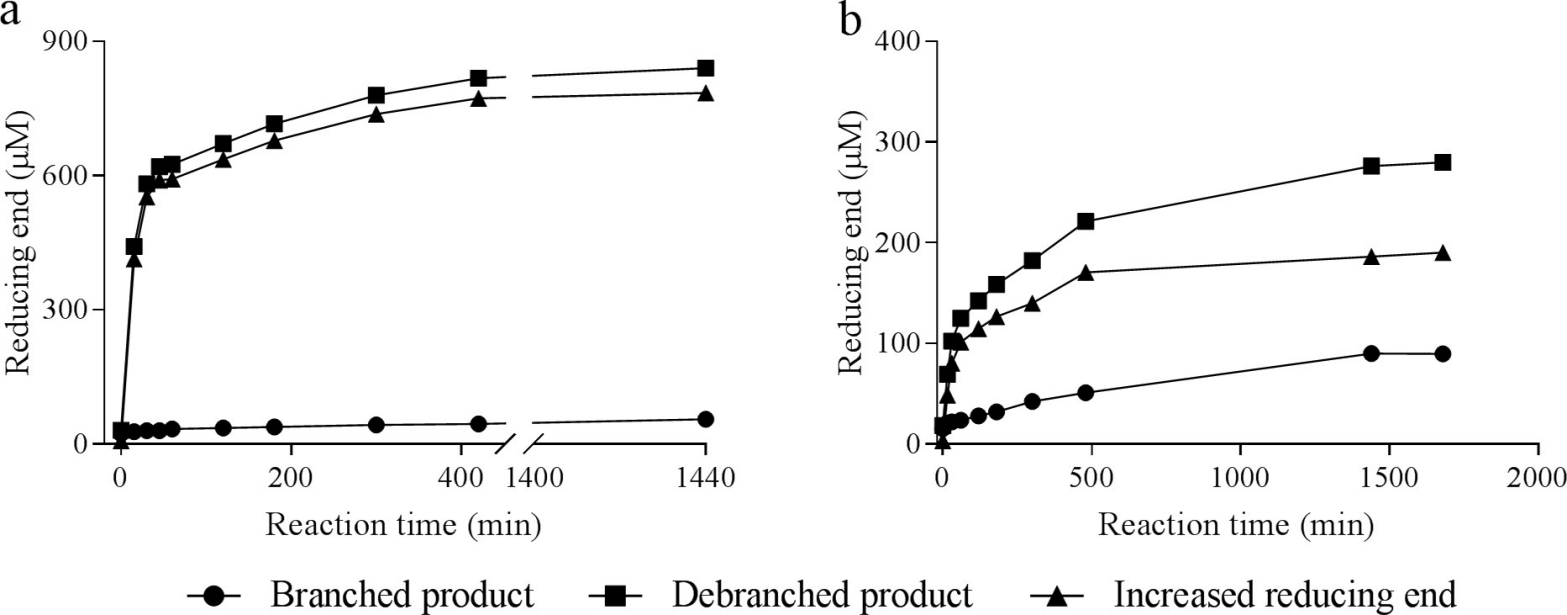
Increase in reducing end following the branching and hydrolytic activity in time.

GBEs create branches via a transglycosylation reaction, in which a new α-1,4-gluco-oligosaccharide chain is used as an acceptor. A side reaction of GBEs is hydrolysis, in which water is used as an acceptor, resulting in the formation of short α-1,4-glucan chains. The branching and hydrolysis reaction of both GBEs with amylose V as substrate was followed in time (Fig 4). The use of amylose V as substrate has the advantage that it is virtually free from α-1,6-linkages, so any α-1,6-bond present in the product is the result of the branching activity of the GBE. PmGBE13 rapidly branches amylose V into a branched α-glucan within the first 50 min of the reaction (Fig 4A). Differently, the PmGBE57 is clearly slower in introducing α-1,6-linkages (Fig 4B) when given amylose. The branching activity of PmGBE13 and PmGBE57 is 6 U/mg protein and 0.04 U/mg protein, respectively. The branching activity of PmGBE13 is similar to that reported for the GH13 GBE from *Deinococcus geothermalis* [44], and relatively high compared to the previously reported GBEs from *E*. *coli*, *Aquifex aeolicus*, *Geobacillus stearothermophilus* and *Anaerobranca gottschalkii* [45–48]. The PmGBE57 showed relatively lower activity than GH57 GBEs from *T*. *thermophilus*, *Pyrococcus horikoshii* [26, 28]. The hydrolytic activity of PmGBE13 and PmGBE57 is 0.006 U/mg and 0.003 U/mg, being comparable to the hydrolytic activity of the GBE of *D*. *geothermalis* [44], and *T*. *thermophilus* [28], resp.

### Structural properties of the branched α-glucans

**Fig 5:**
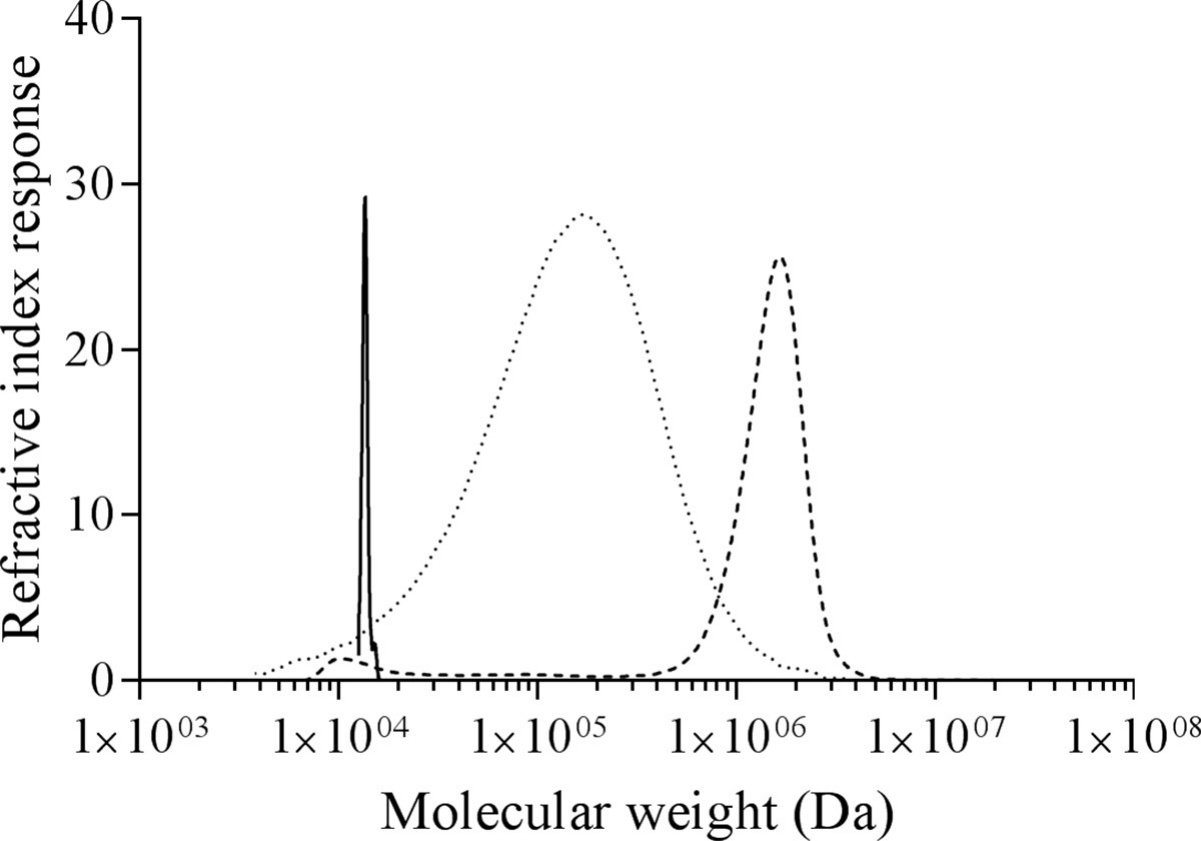
Molecular size distribution of amylose V (.....) and the branched α-glucan products made by PmGBE13 (----) and PmGBE57 (-).

PmGBE13 made branched α-glucans with an average molecular mass of 1.7×10^6^ Da, which is 8.5 times larger than that of amylose V, having an average molecular mass of 2×10^5^ Da (Fig 5). The PmGBE13 product shows a relatively high molecular mass compared to GBE modified waxy corn starch [37], while it is lower than the glycogen from *Sphaerotilus natans*, *Arthrobacter viscous* and oyster [49]. On the other hand, PmGBE57 made a branched α-glucan that was considerably smaller than amylose V; the average molecular mass was 1.4×10^4^ Da (approx. 85 glucose units), which is much smaller than glycogen produced by bacteria, and even 10 times smaller than the glycogen-like carbohydrate polymers extracted from the cell wall of *M*. *tuberculosis* [50, 51].

**Fig 6:**
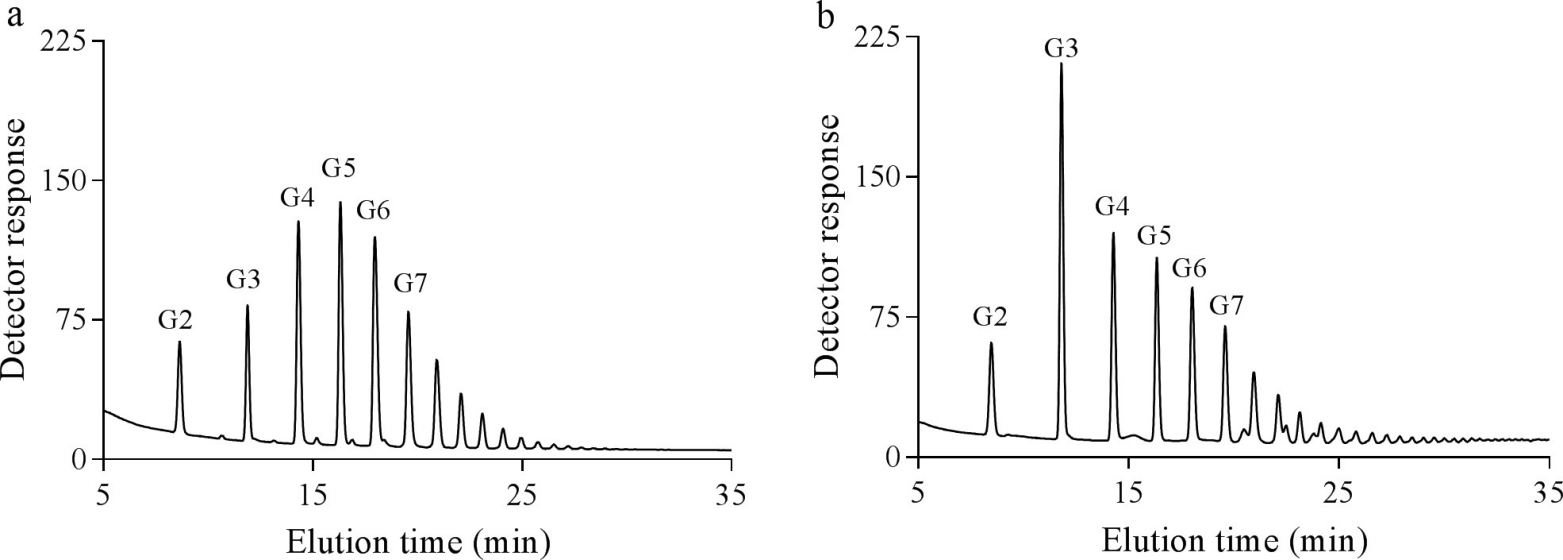
**The chain length distribution of the branched α-glucans derived from amylose V incubated with PmGBE13 (a) and PmGBE57 (b) in phosphate buffer pH 7.0 at 50°C for 24 h**.

To further elucidate the structure of the branched α-glucans, the chain length distribution, average chain length (ACL), and average internal chain length (AICL) were determined (Table 2). The PmGBE13 product has side chains ranging from 2 to 15 residues, showing a bell-shaped distribution (Fig 6A); the ACL is 8 and the AICL is 2.6. The PmGBE57 product has, in contrast, considerable shorter side chains of 3 to 5 residues (Fig 6B). The ACL of PmGBE57 is 7 while the AICL is DP 2.4. Although the ACL and AICL of the PmGBE13 and PmGBE57 products did not differ significantly, a clear difference was found for the percentage of A-chains in the PmGBE13 and PmGBE57 products; the PmGBE13 product contained 28% A-chains while the PmGBE57 product contained more than 44% A-chains (Table 2). All these results taken together show that PmGBE13 makes a completely different branched α-glucan than PmGBE57, hinting at the involvement of the two enzymes in different biosynthetic pathways or the presence of two structurally different intracellular branched α-glucans in *P*. *mobilis*.

**Table 2:** Chain length distribution, average chain length (ACL), average internal chain length (AICL), and A-chain content of PmGBE13 and PmGBE57 branched α-glucans from amylose.

	PmGBE13	PmGBE57
∑ DP 2–4 (%)	19	28
∑ DP 5–10 (%)	75	59
∑ DP > 10 (%)	6	13
ACL (DP)	8	7
AICL (DP)	2.6	2.4
A-chain (%)	28	44

### Minimal donor substrate length

**Fig 7:**
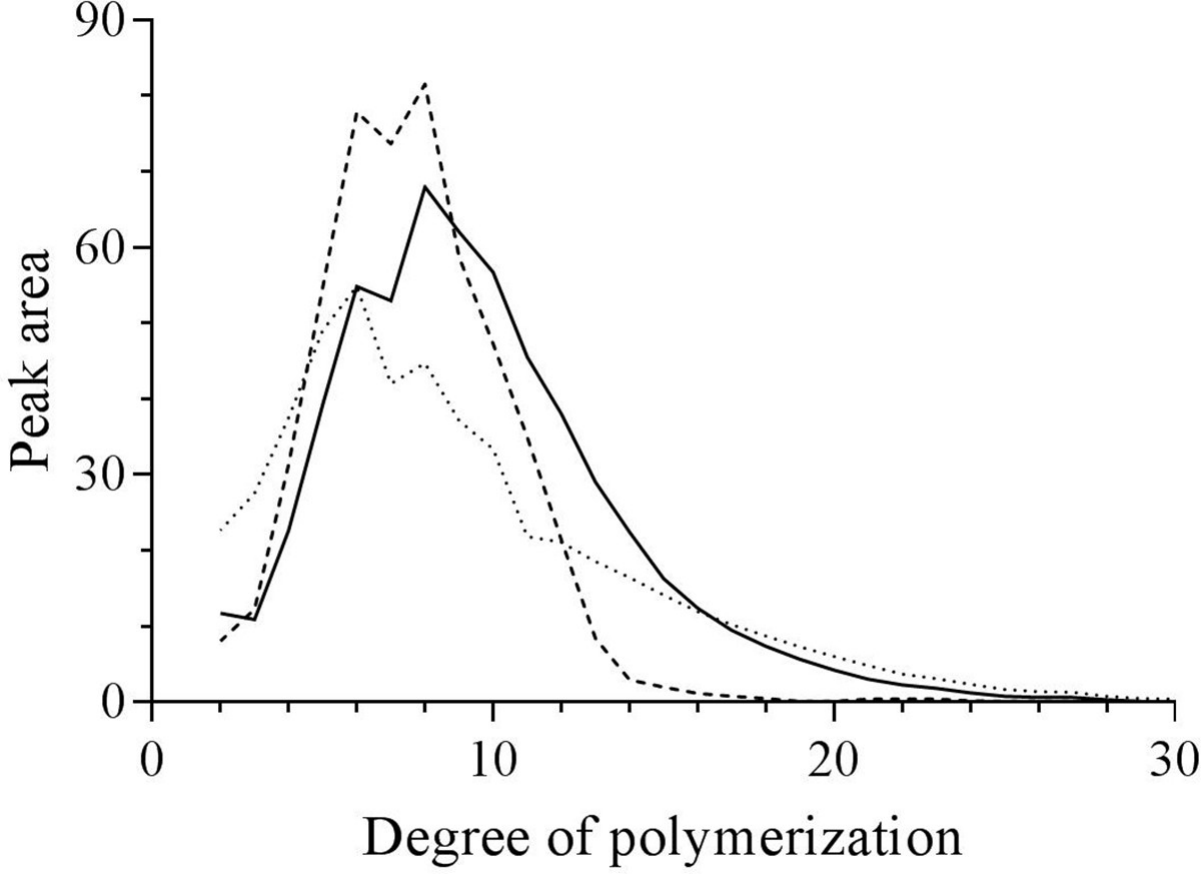
The chain length distribution of the products derived when debranched HBS (....) was incubated with PmGBE13 (----) or PmGBE57 (-) at 50°C for 24 h.

GBEs require a minimum length of substrate before a branching or hydrolysis reaction can start [45]. As such, the minimum chain length of the donor substrate is an important parameter of GBEs. To assess this specificity, PmGBE13 and PmGBE57 were incubated with a mixture of linear chains in the DP range of 2 to 30 (DHBS). Subsequent analysis of the debranched products showed that PmGBE13 had converted all linear oligosaccharides with a DP of 13 and more (Fig 7). PmGBE57 requires slightly longer linear oligosaccharides, as it had converted all linear oligosaccharides of DP 17 and longer (Fig 7). The PmGBE13 branched α-glucan product is rich in side chains with 5 and 6 residues while the PmGBE57 branched glucan product is composed of longer chains with a maximum at 9 to 10 residues (Fig 7), this being in line with the results found with amylose V as substrate (Fig 3). Thus, the minimum substrate length for PmGBE13 is 13 residues, this being very close to what was found for the GH13 GBEs of *Rhodothermus obamensis* and *E*. *coli* that use donor substrates of minimally 12 residues [27, 45]. For GH57 GBEs, no minimum substrate lengths have been published so far.

### Role of GH13 and GH57 GBEs

The human pathogen *M*. *tuberculosis* has two *glgB* genes, one encoding a GH13 GBE (Rv1326c) and another encoding a GH57 GBE (Rv3031). A knock-out of the *glgB* gene Rv1326c in *M*. *tuberculosis* H37Rv did not give any viable mutants. The knock-out mutation could be compensated by a plasmid carrying either the *gbe* gene from *M*. *tuberculosis* or *E*. *coli*, demonstrating that the *gbe* gene Rv1326c and its corresponding enzyme are essential for growth [22]. It remains unclear what the role of Rv3031 in *M*. *tuberculosis* is. Apparently Rv3031 does not take over the role of Rv1362c in glycogen biosynthesis, either because the gene was not expressed in the Rv1326c knock-out mutant or because the GH57 GBE enzyme does not act on a growing linear α-glucan chain as the GH13 GBE does [21]. The Rv3031 gene has been linked to the synthesis of the capsular glucans typical for *M*. *tuberculosis*, although without experimental evidence [52, 53]. The results reported in this paper show that the GH13 and the GH57 GBE of *P*. *mobilis* differ considerably with respect to activity towards amylose and the structure of the branched α-glucan produced. The high molecular mass of the branched α-glucan product points at a role for the PmGBE1 in glycogen biosynthesis as glycogen is a large molecule of 10^6^ to 10^7^ Da [49], this being in line with the role Rv1326c plays in glycogen production in *M*. *tuberculosis* [22]. The role of PmGBE2 as is the role of the GH57 GBE in *M*. *tuberculosis* remains unclear and calls for further investigation.

## Conclusions

The majority of the genome sequences of *Petrotogaceae* harbor two *glgB* genes, encoding a GH13 and a GH57 GBE. Both genes have all features to encode the corresponding proteins and overexpression in *E*. *coli* resulted in active GBEs. The lack of a clearly recognizable signal sequence and the activity at neutral pH point at an intracellular localization of both enzymes. The PmGH13 seems a common GBE with a high activity and synthesizing highly branched and relative high MW α-glucans of 10^6^–10^7^ Da. The PmGBE57, in contrast has a very low branching activity with amylose as substrate and forms branched α-glucans of considerably lower MW (10^4^ Da) with a lower degree of branching making it very unlikely that this enzyme plays a role in glycogen biosynthesis. Further studies including detailed analysis of the structure of *Petrotoga* glycogen structure, gene expression, and gene knock-outs should shed some light on the role of the GH13 and GH57 GBE during the biosynthesis of glycogen and/or other α-glucans in *P*. *mobilis* and other *Petrotogacaea*.

## Supporting information

S1 Appendix Gene sequence of *glgB13*. (DOCX)

S2 Appendix Gene sequence of *glgB57*. (DOCX)

## References

[1] Itoh T, Onishi M, Kato S, Iino T, Sakamoto M, Kudo T, et al *Athalassotoga saccharophila* gen. nov., sp nov., isolated from an acidic terrestrial hot spring, and proposal of *Mesoaciditogales* ord. nov and *Mesoaciditogaceae* fam. nov in the phylum *Thermotogae*. Int J Syst Evol Micr. 2016;66:1045–1051. 10.1099/ijsem.0.000833 26651491

[2] Bhandari V, Gupta RS. Molecular signatures for the phylum (class) *Thermotogae* and a proposal for its division into three orders (*Thermotogales*, *Kosmotogales* ord. nov and *Petrotogales* ord. nov.) containing four families (*Thermotogaceae*, *Fervidobacteriaceae* fam. nov., *Kosmotogaceae* fam. nov and *Petrotogaceae* fam. nov.) and a new genus *Pseudothermotoga* gen. nov with five new combinations. Anton Leeuw Int J G. 2014;105(1):143–168. 10.1007/s10482-013-0062-7 24166034

[3] Huber R, Woese CR, Langworthy TA, Fricke H, Stetter KO. *Thermosipho africanus* Gen-Nov, represents a new genus of thermophilic eubacteria within the Thermotogales. Syst Appl Microbiol. 1989;12(1):32–37. 10.1016/S0723-2020(89)80037-2

[4] DiPippo JL, Nesbo CL, Dahle H, Doolittle WF, Birkland NK, Noll KM. *Kosmotoga olearia* gen. nov., sp nov., a thermophilic, anaerobic heterotroph isolated from an oil production fluid. Int J Syst Evol Micr. 2009;59:2991–3000. 10.1099/ijs.0.008045-0 19643902

[5] Reysenbach AL, Liu YT, Lindgren AR, Wagner ID, Sislak CD, Mets A, et al *Mesoaciditoga lauensis* gen. nov., sp nov., a moderately thermoacidophilic member of the order *Thermotogales* from a deep-sea hydrothermal vent. Int J Syst Evol Micr. 2013;63:4724–4729. 10.1099/ijs.0.050518-0 23959829

[6] Nesbo CL, Bradnan DM, Adebusuyi A, Dlutek M, Petrus AK, Foght J, et al *Mesotoga prima* gen. nov., sp nov., the first described mesophilic species of the *Thermotogale*. Extremophiles: life under extreme conditions. 2012;16(3):387–393. 10.1007/s00792-012-0437-0 22411358

[7] Davey ME, Wood WA, KEy R, Nakamura K, Stahl DA. Isolation of three species of *Geotoga* and *Petrotoga*: two new genera, representing a new lineage in the bacterial line of descent distantly related to the “Thermotogales”. Syst Appl Microbiol. 1993;16(2):191–200. 10.1016/S0723-2020(11)80467-4

[8] Ben Hania W, Godbane R, Postec A, Hamdi M, Ollivier B, Fardeau ML. *Defluviitoga tunisiensis* gen. nov., sp nov., a thermophilic bacterium isolated from a mesothermic and anaerobic whey digester. Int J Syst Evol Micr. 2012;62:1377–1382. 10.1099/ijs.0.033720-0 21828011

[9] Wery N, Lesongeur F, Pignet P, Derennes V, Cambon-Bonavita MA, Godfroy A, et al *Marinitoga camini* gen, nov., sp nov., a rod-shaped bacterium belonging to the order *Thermotogales*, isolated from a deep-sea hydrothermal vent. Int J Syst Evol Micr. 2001;51:495–504. 10.1099/00207713-51-2-495 11321096

[10] Jayasinghearachchi HS, Lal B. *Oceanotoga teriensis* gen. nov., sp. nov., a thermophilic bacterium isolated from offshore oil-producing wells. Int J Syst Evol Micr. 2011;61:554–560. 10.1099/ijs.0.018036-0 20382783

[11] Preiss J. Bacterial glycogen synthesis and its regulation. Annu Rev Microbiol. 1984;38:419–458. 10.1146/annurev.mi.38.100184.002223 6093684

[12] Strange RE, Ness AG, Dark FA. Survival of stationary phase *Aerobacter aerogenes* stored in aqueous suspension. J Gen Microbiol. 1961;25(1):61–76. 10.1099/00221287-25-1-61

[13] Burklen L, Schock F, Dahl MK. Molecular analysis of the interaction between the *Bacillus subtilis* trehalose repressor TreR and the tre operator. Mol Gen Genet. 1998;260(1):48–55. 10.1007/s004380050869 9829827

[14] Dinadayala P, Sambou T, Daffe M, Lemassu A. Comparative structural analyses of the α-glucan and glycogen from *Mycobacterium bovis*. Glycobiology. 2008;18(7):502–8. 10.1093/glycob/cwn031 18436565

[15] Thevelein JM, Hohmann S. Trehalose synthase—Guard to the gate of glycolysis in yeast. Trends Biochem Sci. 1995;20(1):3–10. 10.1016/S0968-0004(00)88938-0 7878741

[16] Boyer CD, Preiss J. Starch branching enzymes from developing kernels of maize (Zea-Mays-L). Plant Physiol. 1977;59(6):5–5.

[17] Janecek S, Blesak K. Sequence-structural features and evolutionary relationships of family GH57 α-amylases and their putative α-amylase-like homologues. Protein J. 2011;30(6):429–435. 10.1007/s10930-011-9348-7 21786160

[18] Blesak K, Janecek S. Sequence fingerprints of enzyme specificities from the glycoside hydrolase family GH57. Extremophiles: life under extreme conditions. 2012;16(3):497–506. 10.1007/s00792-012-0449-9 22527043

[19] Suzuki E, Suzuki R. Distribution of glucan branching enzymes among prokaryotes. Cell Mol Life Sci. 2016;73(14):2643–2660. 10.1007/s00018-016-2243-9 27141939 PMC11108348

[20] Murakami T, Kanai T, Takata H, Kuriki T, Imanaka T. A novel branching enzyme of the GH-57 family in the hyperthermophilic archaeon *Thermococcus kodakaraensis* KOD1. J Bacteriol. 2006;188(16):5915–5924. 10.1128/JB.00390-06 16885460 PMC1540076

[21] Garg SK, Alam MS, Kishan KR, Agrawal P. Expression and characterization of α-(1, 4)-glucan branching enzyme Rv1326c of *Mycobacterium tuberculosis* H37Rv. Protein Expres Purif. 2007;51(2):198–208. 10.1016/j.pep.2006.08.005 17005418

[22] Sambou T, Dinadayala P, Stadthagen G, Barilone N, Bordat Y, Constant P, et al Capsular glucan and intracellular glycogen of *Mycobacterium tuberculosis*: biosynthesis and impact on the persistence in mice. Mol Microbiol. 2008;70(3):762–774. 10.1111/j.1365-2958.2008.06445.x 18808383 PMC2581643

[23] Chandra G, Chater KF, Bornemann S. Unexpected and widespread connections between bacterial glycogen and trehalose metabolism. Microbiol-Sgm. 2011;157:1565–1572. 10.1099/mic.0.044263-0 21474533

[24] Mendes V, Maranha A, Alarico S, Empadinhas N. Biosynthesis of mycobacterial methylglucose lipopolysaccharides. Nat Prod Rep. 2012;29(8):834–844. 10.1039/c2np20014g 22678749

[25] Boyer C, Preiss J. Biosynthesis of bacterial glycogen—Purification and properties of *Escherichia coli* B α-1,4-glucan-α-1,4-glucan 6-glycosyltransferase. Biochemistry. 1977;16(16):3693–3699. 10.1021/bi00635a029 407932

[26] Na S, Park M, Jo I, Cha J, Ha NC. Structural basis for the transglycosylase activity of a GH57-type glycogen branching enzyme from *Pyrococcus horikoshii*. Biochem Bioph Res Co. 2017;484(4):850–856. 10.1016/j.bbrc.2017.02.002 28163025

[27] Roussel X, Lancelon-Pin C, Vikso-Nielsen A, Rolland-Sabate A, Grimaud F, Potocki-Veronese G, et al Characterization of substrate and product specificity of the purified recombinant glycogen branching enzyme of *Rhodothermus obamensis*. Bba-Gen Subjects. 2013;1830(1):2167–2177. 10.1016/j.bbagen.2012.09.022 23041072

[28] Palomo M, Pijning T, Booiman T, Dobruchowska JM, van der Vlist J, Kralj S, et al *Thermus thermophilus* glycoside hydrolase family 57 branching enzyme crystal strucutre, mechanism of action, and products formed. J Biol Chem. 2011;286(5):3520–3530. 10.1074/jbc.M110.179515 21097495 PMC3030357

[29] Cantarel BL, Coutinho PM, Rancurel C, Bernard T, Lombard V, Henrissat B. The Carbohydrate-Active EnZymes database (CAZy): an expert resource for Glycogenomics. Nucleic Acids Res. 2009;37:D233–D238. 10.1093/nar/gkn663 18838391 PMC2686590

[30] Guan HP, Preiss J. Differentiation of the properties of the branching isozymes from maize. Plant Physiol. 1993;102(4):1269–1273. 10.1104/pp.102.4.1269 12231902 PMC158914

[31] Waffenschmidt S, Jaenicke L. Assay of reducing sugars in the nanomole range with 2,2'-bicinchoninate. Anal Biochem. 1987;165(2):337–340. 10.1016/0003-2697(87)90278-8 3425902

[32] van Leeuwen SS, Leeflang BR, Gerwig GJ, Kamerling JP. Development of a H^1^ NMR structural-reporter-group concept for the primary structural characterisation of α-D-glucans. Carbohyd Res. 2008;343(6):1114–1149. 10.1016/j.carres.2008.01.043 18314096

[33] Peat S, Whelan WJ, Thomas GJ. Evidence of multiple branching in waxy maize starch. J Chem Soc. 1952;(11):4546–4548. 10.1016/0006-291X(78)91119-1

[34] Thompson DB. On the non-random nature of amylopectin branching. Carbohyd Polym. 2000;43(3):223–239. 10.1016/S0144-8617(00)00150-8

[35] Robyt JF. Enzymes and their action on starch. Starch (Third Edition): Elsevier; 2009 p. 237–292.

[36] Preiss J. Bacterial glycogen-synthesis and yts regulation. Annu Rev Microbiol. 1984;38:419–458. 10.1146/annurev.mi.38.100184.002223 6093684

[37] Lee B-H, Yan L, Phillips RJ, Reuhs BL, Jones K, Rose DR, et al Enzyme-synthesized highly branched maltodextrins have slow glucose generation at the mucosal α-glucosidase level and are slowly digestible in vivo. Plos One. 2013;8(4):e59745 10.1371/journal.pone.0059745 23565164 PMC3615069

[38] Fan Q, Xie Z, Zhan J, Chen H, Tian Y. A glycogen branching enzyme from *Thermomonospora curvata*: Characterization and its action on maize starch. Starch-Stärke. 2016;68(3–4):355–364. 10.1002/star.201500197

[39] Dinadayala P, Sambou T, Daffé M, Lemassu A. Comparative structural analyses of the α-glucan and glycogen from *Mycobacterium bovis*. Glycobiology. 2008;18(7):502–508. 10.1093/glycob/cwn031 18436565

[40] Liu Y, Li C, Gu Z, Xin C, Cheng L, Hong Y, et al Alanine 310 is important for the activity of 1, 4-α-glucan branching enzyme from *Geobacillus thermoglucosidans* STB02. Int J Biol Macromol. 2017;97:156–163. 10.1016/j.ijbiomac.2017.01.028 28082221

[41] Van Der Maarel MJ, Van der Veen B, Uitdehaag JC, Leemhuis H, Dijkhuizen LJJob. Properties and applications of starch-converting enzymes of the α-amylase family. 2002;94(2):137–155. 10.1016/s0168-1656(01)00407-2 11796168

[42] Zhang GY, Hasek LY, Lee BH, Hamaker BR. Gut feedback mechanisms and food intake: a physiological approach to slow carbohydrate bioavailability. Food Funct. 2015;6(4):1072–1089. 10.1039/c4fo00803k 25686469

[43] Kittisuban P, Lee BH, Suphantharika M, Hamaker BR. Slow glucose release property of enzyme synthesized highly branched maltodextrins differs among starch sources. Carbohyd Polym. 2014;107:182–191. 10.1016/j.carbpol.2014.02.033 24702934

[44] Palomo M, Kralj S, van der Maarel MJEC, Dijkhuizen L. The unique branching patterns of *Deinococcus* glycogen branching enzymes are determined by their N-terminal domains. Appl Environ Microb. 2009;75(5):1355–1362. 10.1128/Aem.02141-08 19139240 PMC2648161

[45] Guan HP, Li P, ImparlRadosevich J, Preiss J, Keeling P. Comparing the properties of Escherichia coli branching enzyme and maize branching enzyme. Arch Biochem Biophys. 1997;342(1):92–98. 10.1006/abbi.1997.0115 9185617

[46] Takata H, Takaha T, Kuriki T, Okada S, Takagi M, Imanaka T. Properties and active-center of the thermostable branching enzyme from *Bacillus stearothermophilus*. Appl Environ Microb. 1994;60(9):3096–3104. 10.1128/aem.60.9.3096-3104.1994 PMC201776 7944355

[47] Thiemann V, Saake B, Vollstedt A, Schafer T, Puls J, Bertoldo C, et al Heterologous expression and characterization of a novel branching enzyme from the thermoalkaliphilic anaerobic bacterium *Anaerobranca gottschalkii*. Appl Microbiol Biotechnol. 2006;72(1):60–71. 10.1007/s00253-005-0248-7 16408175

[48] Van der Maarel MJEC, Vos A, Sanders P, Dijkhuizen L. Properties of the glucan branching enzyme of the hyperthermophilic bacterium *Aquifex aeolicus*. Biocatal Biotransfor. 2003;21(4–5):199–207. 10.1080/10292920310001618528

[49] Martinez-Garcia M, Stuart MCA, van der Maarel MJE. Characterization of the highly branched glycogen from the thermoacidophilic red microalga *Galdieria sulphuraria* and comparison with other glycogens. Int J Biol Macromol. 2016;89:12–18. 10.1016/j.ijbiomac.2016.04.051 27107958

[50] Lemassu A, Daffé M. Structural features of the exocellular polysaccharides of *Mycobacterium tuberculosis*. Biochem J. 1994;297(2):351–357. 10.1042/bj2970351 8297342 PMC1137836

[51] Ortalo-Magne A, Dupont M-A, Lemassu A, Andersen AB, Gounon P, Mamadou D. Molecular composition of the outermost capsular material of the tubercle bacillus. Microbiology. 1995;141(7):1609–1620. 10.1099/13500872-141-7-1609 7551029

[52] Kaur D, Guerin ME, Škovierová H, Brennan PJ, Jackson MJAiam. Biogenesis of the cell wall and other glycoconjugates of *Mycobacterium tuberculosis*. 2009;69:23–78. 10.1016/S0065-2164(09)69002-X PMC3066434 19729090

[53] Stadthagen G, Sambou T, Guerin M, Barilone N, Boudou F, Korduláková J, et al Genetic basis for the biosynthesis of methylglucose lipopolysaccharides in *Mycobacterium tuberculosis*. 2007;282(37):27270–27276. 10.1074/jbc.M702676200 17640872

