# Supplementary material for: Characterization of the GH13 and GH57 glycogen branching enzymes from Petrotoga mobilis SJ95 and potential role in glycogen biosynthesis
Source: PLoS One. 2019 Jul 15;14(7):e0219844. doi: 10.1371/journal.pone.0219844 (PMC6629080; doi:10.1371/journal.pone.0219844)
Supplement: S1 Appendix — (DOCX) [file pone.0219844.s001.docx]

**Supporting information**

**S1 Appendix. Gene sequence of *glgB13.***

ATGCCGATTCTGACCGAGAAAGAAATCAACATGCTGGTGAACGCGGAATGCCACGATCCGTTCATCTATCTGGGT

CTGCGTGAACTGGACAAGGAGAACCTGGTGATTCGTGTTCTGCAGCCGTTTGCGAAAGAAGCGTACCTGATCAGC

AAGAACACCACCATCAAGCTGGATAAAATTCACAAGACCGGTCTGTTCGAGAAGAAAATCCCGGGCAGCCAAATT

TTTGAGTATGAAATCAAATGCACCGATTACAACGCGAACACCTGGACCTATAAGGACCCGTACAGCTTCCTGCCG

GTTATTAGCGAATATGACCGTTACCTGTTTAACGAGGGTAACCACTATAAAATCTACGAAAAGCTGGGCGCGCAC

CCGATGAAAATCAACGGTATTGAGGGCGTGCTGTTCAGCACCTGGGCGCCGAACGCGAAGCGTGTTAGCGTGGTT

GGTAACTTTAACAACTGGGATGGCCGTATCCACCAGATGCGTGTGCTGGGTCAAAGCGGCATCTGGGAAATTTTC

GTTCCGGGTGTGGTTGAAGGCGACCTGTACAAATTTGAGATTAAGACCCAGAACGGTGATATCCTGCTGAAAACC

GACCCGTATGGCACCTACTTCGAAGTGCGTCCGAAGACCGCGGCGATCGTTTATGATAAACAAAACAAGCACACC

TGGAACGACAGCAAATGGATGGAGGAGCGTAAGAACAAAAACTGGATTAAGGAACCGATGAGCATCTACGAGGTG

CACCTGGGTAGCTGGGCGCGTAAAGAGGAAAACCAGTTCCTGAACTATCGTGAACTGGCGGAGAAACTGAGCGCG

TACCTGAAGGAAAACCACTTTACCCACATTGAACTGCTGCCGGTGAGCGAGCACCCGCTGGATATCAGCTGGGGT

TATCAGGTTACCGGCTACTATGCGCCGACCAGCCGTTTCGGTAAACCGGAAGATTTCATGTACTTTGTGGACAAG

ATGCACCAAGAGGGTATCGGCGTGATTGTTGACTGGGTTCCGGGCCACTTCCCGAAAGATGCGCACGCGCTGGGT

CGTTTTGACGGCACCGCGCTGTACGAGCACCTGGATAGCCGTCTGGGTGAACACAAGGACTGGGGCACCTATATC

TTTAACTACGGTCGTACCGAAGTGAAAAACTTCCTGCTGGCGAACGCGCTGTTTTGGCTGGACAAGTATCACATT

GATGGTCTGCGTGTGGACGCGGTTGCGAGCATGCTGTATCTGGATTACAGCCGTCAGGACGGCGAGTGGATTCCG

AACAAATACGGTGGCCGTGAAAACCTGGAGGCGATCGACTTCATTAAGCAGCTGAACACCGTTGCGTACCAATAT

TTTCCGGGTATTGTGACCGTTGCGGAGGAAAGCACCGCGTTTCCGGGCGTGACCACCCCGGTTCATGAAGGTGGC

CTGGGTTTCGAGTTTAAATGGAACATGGGCTGGATGAACGATACCCTGCGTTATTTCAGCAAGGACCCGATCTTT

CGTCGTTACCACCAAAACGATCTGACCTTTAGCCTGGTTTACGCGTTCAGCGAGAACTTTATCCTGAGCATTAGC

CACGACGAAGTGGTTCACGGTAAACGTAGCCTGCTGGAGAAGATGCCGGGCGACGATTGGCAGAAGTTCGCGAAC

CTGCGTCTGTTTTACAGCTATATGTACGCGCACCCGGGTAAAAACCTGCTGTTCATGGGCAGCGAAATTGCGCAG

CGTAAGGAGTGGGATTGCAGCAACAGCCTGGACTGGCACCTGCTGCAGTACGAACCGCACAAGAAAACCCAACTG

TTCCTGAAAGATCTGAACATCCTGTATAAGAACCACCCGGCGCTGTTTGAGATTGATCACCGTTACGAAGGTTTC

GAGTGGATCGACTTTAACGACGCGGATAACAGCGTGATCAGCTTCATTCGTAAAAGCGAGAACGAAGATGAGATC

ATTGTGTGCGTTTTCAACTTTACCCCGGTTCCGCGTGACAACTATCGTATTGGTGTGCCGAAGAAAGGCGTTTAC

CAGGAGATCCTGAACACCGACTGGACCCAATACTATGGTAGCGGCGTGGATAACCCGAAGGAAGTTTATAGCCAG

GACATTCCGATGCACGGCCGTGAGCAAAGCATCATTCTGACCCTGCCGCCGCTGGGTGGCCTGTACTTCAAATTT
CTGATCAAG

**S2 Appendix. Gene sequence of *glgB57.***

ATGAAGAGCCGTAAGGGTAAAATCCTGTTTCTGCTGCACGCGCACCTGCCGTACATTCACCACCCGGATTTCGAG

AACTTTATGGAGGAACGTTGGTTCTTTGAAGCGCTGACCGAAACCTATATCCCGCTGATTAAGGTGTTTAAAAGC

CTGGAAAAGGATAAAATCCCGTTCAAGCTGACCATTAGCCTGAGCCCGACCCTGATGGAAATGTTCAACCTGAAA

GACCTGCGTGAGAAGTACCACAAATATCTGCTGAACCTGATCGAACTGACCGAAAAGGAGATCATTCGTACCAAA

GACGAGGACCCGCGTATCCACCAGCTGGCGCAACACTACCGTCGTGAACTGATCGAGGACCTGGATATCTTCTGC

GAGGAATATAACCAGAACATCCTGAAGGCGTTCAAGGAATTCAAGGATAAGGGTTACATCGAAGTGATTACCAGC

AACGGTACCCACGGCTATCTGCCGTTCTACCGTGACTATCCGGAGGCGATCCGTGCGCAAATTAAAAGCGCGGTT

CTGACCTTCAAGAAAAACTTTGGTGAACATCCGATGGGCATCTGGCTGGCGGAGTGCGCGTACTTTAAGAGCCTG

GACAAATATCTGAGCGATGAAGGTATTCGTTACTTCTTTGTGGGTACCCACGGCTTCACCTATGCGGACAGCCAG

CCGCGTTACGGCGTTTATCGTCCGATCATTACCCCGAACAAGGTGTTCGTTTTTGCGCGTGATCCGGAAAGCAGC

GAGCAAATCTGGAGCAGCGAGGTGGGTTACCCGGGTGACAGCCGTTACCGTGAATTCTATCGTGACATCGGCTAT

GATCGTGAGGACGATTACATCAAACCGTATATTGATCCGAGCGGTACCCGTTGCAACACCGGCATCAAGTACCAC

CGTATTACCGACAAAAGCCTGAGCCTGGATAAGAAAGAAATCTATGACCTGCGTGAGGCGCGTAACGCGGTGAAG

GAACACGTTAAGGACTTCATCTTCAAGAAAACCAGCCAGATCCGTAAGCTGGCGGCGATTCTGGATGAGGAAGAG

CCGATCATTGTGGCGCCGTTCGACGCGGAACTGTTTGGTCACTGGTGGTACGAGGGCCCGAAATTCCTGGAAGAG

CTGTTTCGTCAAAGCAGCGAAAACGAGTATCTGGATTTTAGCGTTCCGACCGAAATCCTGCAGACCGTGAAGAAA

GTTCAAATTACCTATCCGGCGGAGAGCAGCTGGGGTGCGGGTGGCTATCACGATGTGTGGCTGAACGAAAAGAAC

GACTGGGTTTACAAACACATCCACGAAATTACCGAGCGTATGATCGAGAAGGCGAACACCTTCAAAAACCCGAGC

AACCTGCAGAAGCGTGCGCTGAACCAAATGATGCGTGAGGTTCTGCTGGCGCAGGCGAGCGACTGGCCGTTTATC

ATGACCACCGGCACCACCATTGAATACGCGAAGAACCGTGTGAAATGCCACATCAACCGTTTCCTGGACCTGGAT

AAGATGCTGGAAAAACAGCAAGTTAACGAAGAGCGTCTGAGCTTCTACGAGTGGATCGACGATATCTTCCGTAAC
ATCGATTACACCATTTTCAGCAGCGACTATCGTACCAAC
